# CVD Synthesis of MoS_2_ Using a Direct MoO_2_ Precursor: A Study on the Effects of Growth Temperature on Precursor Diffusion and Morphology Evolutions

**DOI:** 10.3390/ma16134817

**Published:** 2023-07-04

**Authors:** Ratchanok Somphonsane, Tinna Chiawchan, Waraporn Bootsa-ard, Harihara Ramamoorthy

**Affiliations:** 1 Department of Physics, School of Science, King Mongkut’s Institute of Technology Ladkrabang, Bangkok 10520, Thailand; ratchanok.so@kmitl.ac.th (R.S.); 62605032@kmitl.ac.th (T.C.); 62050723@kmitl.ac.th (W.B.-a.); 2 Thailand Center of Excellence in Physics, Commission on Higher Education, 328 Si Ayutthaya Road, Bangkok 10400, Thailand; 3 Department of Electronics Engineering, School of Engineering, King Mongkut’s Institute of Technology Ladkrabang, Bangkok 10520, Thailand

**Keywords:** two-dimensional (2D) materials, monolayer MoS_2_, chemical vapor deposition, Raman, MoO_2_ precursors, S:Mo ratio, diffusion

## Abstract

In this study, the influence of growth temperature variation on the synthesis of MoS_2_ using a direct MoO_2_ precursor was investigated. The research showed that the growth temperature had a strong impact on the resulting morphologies. Below 650 °C, no nucleation or growth of MoS_2_ occurred. The optimal growth temperature for producing continuous MoS_2_ films without intermediate-state formation was approximately 760 °C. However, when the growth temperatures exceeded 800 °C, a transition from pure MoS_2_ to predominantly intermediate states was observed. This was attributed to enhanced diffusion of the precursor at higher temperatures, which reduced the local S:Mo ratio. The diffusion equation was analyzed, showing how the diffusion coefficient, diffusion length, and concentration gradients varied with temperature, consistent with the experimental observations. This study also investigated the impact of increasing the MoO_2_ precursor amount, resulting in the formation of multilayer MoS_2_ domains at the outermost growth zones. These findings provide valuable insights into the growth criteria for the effective synthesis of clean and large-area MoS_2_, thereby facilitating its application in semiconductors and related industries.

## 1. Introduction

Two-dimensional (2D) MoS_2_ is a popular semiconductor material with a tunable bandgap and high potential for optoelectronic applications [1,2,3]. It has been extensively explored in the literature for transistors, photodetectors, and sensors [4,5,6,7,8,9,10,11,12,13,14,15]. While mechanical exfoliation provides high-quality samples, its limited yield and random crystal sizes pose challenges for practical use. In contrast, chemical vapor deposition (CVD) offers improved scalability, yield, and film homogeneity, making it a promising method for integrating 2D MoS_2_ with existing technologies. However, achieving large-scale, defect-free, and cost-effective production of MoS_2_ remains a crucial goal. CVD-based synthesis of MoS_2_ films has been accomplished through sulfurization of Mo-containing precursors, including Mo [16], MoO_2_ [17,18,19,20,21], and MoO_3_ [22,23,24,25,26,27,28,29,30,31,32,33,34,35,36,37,38,39,40,41,42,43,44,45,46,47,48,49,50,51,52,53,54,55,56,57,58,59]. Among these options, the utilization of MoO_3_ powder as a precursor has emerged as the preferred method, owing to its ability to yield large-area single-crystal films with continuous coverage [42,43,44,45,46,47,48,49,50]. However, it is important to recognize that the use of MoO_3_ powder entails certain drawbacks, namely, its toxic nature (classified as a strong irritant and carcinogen based on GHS standards) and low evaporation temperature (350 °C), which pose significant health hazards and occupational risks. Consequently, it becomes imperative to identify safer alternative precursor materials to ensure that both small-scale research and large-scale production are conducted without compromising health and safety.

The synthesis of MoS_2_ from a conventional MoO_3_ precursor involves a two-step process: reduction of MoO_3_ to MoO_2_, and subsequent sulfurization of MoO_2_ to MoS_2_ [42]. However, incomplete reactions during CVD growth can lead to the formation of a MoS_2−y_O_y_ phase, resulting in phase-mixing with MoS_2_, as frequently observed in experiments [24,26,49,60,61,62]. In a detailed study conducted by Hyun et al. [36], it was concluded that when using MoO_3_ as the precursor, multiple thermodynamically favorable pathways for MoS_2_ synthesis exist, including the sulfurization of vapor-phase MoO_3_, sulfurization of intermediate solid-phase MoO_2_, and sulfurization of vapor-phase MoO_2_. Among these pathways, the direct vapor-phase sulfurization (VPS) of MoO_2_ was found to be the most thermodynamically favorable. In the seminal experimental work of Bilgin et al. [17], an important question was raised as to whether the use of MoO_3_ as a precursor material is really necessary when MoO_2_—a more stable oxide—could be used directly, thereby avoiding the complicated reaction pathways and, hence, enabling a single-step chemical reaction: $MoO_{2}+3S\to MoS_{2}+SO_{2}$. In their work, such a direct, single-step, vapor-phase sulfurization of MoO_2_ was employed to successfully grow MoS_2_ crystals on various substrates.

It is therefore clear that a single-step VPS reaction of MoO_2_ should provide the best chemical pathway to synthesize MoS_2_ after taking into account the associated reaction simplicity, safety, and ability to greatly suppress intermediate-state formations. Despite this, there have been only a few experimental studies [17,18,21] dedicated towards evaluating the potential of directly using MoO_2_ as a precursor. In our recent work [21], we carried out a comprehensive study of the effect of the gas flow rate, MoO_2_ weight, S:Mo molar loading ratio, and growth time on MoS_2_ growth. A high-resolution optical stitching approach was utilized to understand and map the nature of the material grown in various growth zones. We successfully demonstrated that mm-scale continuous films of monolayer MoS_2_, which is free of intermediate states, could be consistently grown provided that the S:Mo loading ratio was kept above the stoichiometrically required value of 3:1 dictated by the VPS reaction of MoO_2_. However, the growth temperature was not varied in that study. Since temperature plays a crucial role in diffusion, it is expected to modify the concentration gradients and, hence, the local S:Mo ratio, leading to strong morphological variations across the growth substrate [21,63].

This work focuses on revealing the effects of growth temperature variation on concentration gradients, growth zone formation, MoS_2_ growth, and the formation of intermediate states. Our results indicate a strong dependence of temperature on the nature of the material grown. We found a cutoff temperature of 600–650 °C, below which nucleation was absent and, therefore, no growth of MoS_2_ was observed. We also observed that the optimal growth temperature, which minimizes intermediate-state formation and maximizes MoS_2_ yield, is approximately 760 °C. XPS analysis of the continuous MoS_2_ revealed good sample quality, with the detection of small amounts of Mo^6+^ states. The stoichiometric ratio of S:Mo was determined using XPS and found to be in close agreement with the expected values. XRD measurements of the CVD-grown material confirmed that the predominant material present was 2H-MoS_2_, with a minor contribution from MoO_2_.

At growth temperatures between 650 and 750 °C, while MoS_2_ can be successfully grown, the yield is poor due to formation of smaller growth zones in this temperature regime. On the other extreme, at growth temperatures exceeding 800 °C, we observed a drastic shift in the nature of the material grown, characterized by a transition from pure MoS_2_ to solely intermediate states grown across the entire substrate. These findings can be explained as a result of a significant increase in precursor diffusion at higher growth temperatures, which results in strong local modifications to the S:Mo loading ratio. By analyzing the classic diffusion equation, we can show qualitatively how the diffusion coefficient, diffusion length, and concentration gradients change as a function of temperature, and how these variations mimic our experimental findings.

The effect of increasing the amount of MoO_2_ precursor was also studied at growth temperatures of 700 °C and 760 °C. The findings revealed the formation of multilayer MoS_2_ domains in the outermost growth zones as the precursor amount was increased. At the ideal growth temperature of 760 °C, we additionally investigated the role of the substrate position. The results demonstrated the formation of the expected parabolic growth zones, with extended growth zones observed downstream, indicating enhanced diffusion in the direction of the gas flow. 

A direct comparison of MoS_2_ growth using MoO_2_ and MoO_3_ under identical experimental conditions revealed a pronounced disparity in the morphology of the resulting materials. This distinction can be attributed to the significant differences in powder vaporization temperatures and the distinct reaction pathways leading to MoS_2_ formation for each precursor. These variations shed light on why the ideal growth conditions can vary significantly depending on the choice of precursor.

The combined findings of this study reveal important growth criteria under which the single-step VPS of MoO_2_ can be effective in growing clean and large-area MoS_2_. With the added advantage of being a safe and scalable approach, this work will enable further developments in the field to successfully employ MoS_2_ as an alternative candidate in the semiconductor and other related industries.

## 2. Materials and Methods

**Figure 1:**
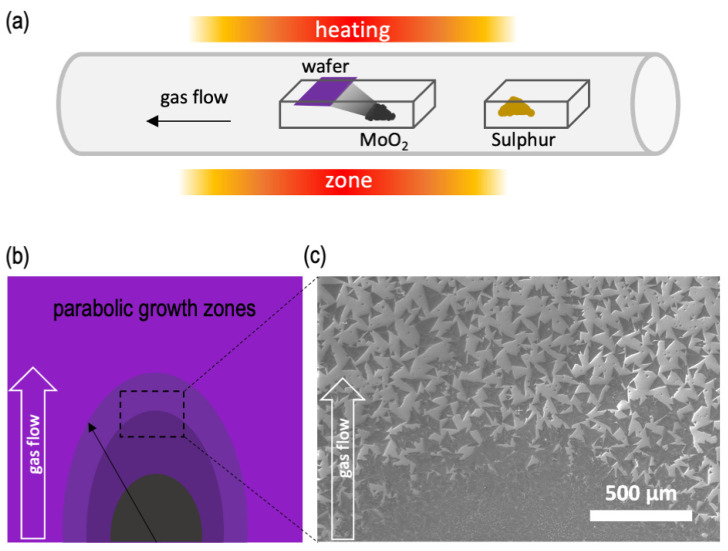
(**a**) Schematic of the CVD setup. The alumina boat containing the MoO_2_ precursor and the growth substrate are positioned at the center of the heating zone, while the S precursor is placed at the edge of the growth zone. The distance between the substrate and the MoO_2_ source is 1 cm. (**b**) Schematic showing the typical zonal growth pattern of MoS_2_; color coding indicates varying morphology in the direction of the gas flow. (**c**) Example low-magnification SEM micrograph capturing the transition from continuous films to individual MoS_2_ domains at the outermost zones indicated (dashed box) in (**b**).

In this study, we synthesized MoS_2_ using the atmospheric-pressure chemical vapor deposition (APCVD) method. Our CVD system consisted of a quartz tube with a diameter of 50 mm and a single heating zone, where MoS_2_ growth occurred at a set growth temperature, which ranged from 600 to 850 °C in this study. Initially, a 300 nm thermal oxide Si/SiO_2_ wafer was cut into approximately 12 mm × 12 mm squares and cleaned by ultrasonication in acetone for 1 min, followed by an isopropyl alcohol (IPA) rinse and nitrogen gas drying. The cleaned wafer, without any additional surface treatment, was then placed face-down at the center of a small alumina boat (40 mm × 8 mm × 7 mm) loaded with MoO_2_ precursor (99% purity Alfa Aesar, Haverhill, MA, USA), positioned approximately 1 cm from the center of the growth substrate (see the schematic in Figure 1a).

Separately, a different quartz boat was loaded with the required amount of sulfur powder (99.5% purity Alfa Aesar, Haverhill, MA, USA) and placed at the edge of the heating zone (details in Figure S1). The tube was then purged with Ar gas (>99.9% purity) at a flow rate of 2000 standard cubic centimeters per minute (sccm) for 5 min at room temperature. Subsequently, the flow rate was adjusted to a low value of 10 sccm, and the temperature of the heating zone was increased at a ramp rate of 15 °C/min until the desired growth temperature was reached, and it was then maintained at this level for a short time of ~1 min. At the end of the growth process, the power to the furnace was turned off, allowing the sample to naturally cool down to room temperature. Typical temperature profiles of the MoO_2_ and S growth zone are shown in Figure S1.

The quality and thickness of the material grown were evaluated through various techniques, including optical imaging, X-ray photoelectron spectroscopy (XPS), micro-Raman spectroscopy, photoluminescence (PL) spectroscopy, scanning electron microscopy (SEM), X-ray diffraction (XRD), and energy-dispersive X-ray spectroscopy (EDS). Optical imaging and high-resolution image stitching were conducted using a CX-40M microscope manufactured by Ningbo Sunny Instruments, Co., Ltd. (Yuyao, China). XPS measurements were performed using an Axis Supra system from Kratos Analytical Ltd., Stretford, Manchester, United Kingdom, with a monochromatic Al Kα (hv = 1486.6 eV) X-ray source. Kratos ESCape software version 1.5 was used for peak assignments, and the core-level spectra were fitted with Gaussian–Lorentzian line shapes after subtracting a Shirley-type background. Stoichiometry calculations were performed by evaluating the relative areas of the relevant components, taking into consideration their system-defined relative sensitivity factors. Micro-Raman and PL spectra were acquired using a Horiba LabRAM HR Evolution confocal Raman system (Horiba Ltd., Bangkok, Thailand) with a laser excitation wavelength of 532 nm. The surface morphology and elemental maps of the grown MoS_2_ films were obtained using a SU8030 Field-Emission Scanning Electron Microscope (FESEM), also from Hitachi High-Tech Ltd. (Bangkok, Thailand). XRD measurements were performed using a Bruker D8 advance system (1.54 Å Cu *Kα* wavelength, 2θ range of 10–90°, 0.02° step size) from Bruker Scientific Instruments, Billerica, MA, USA.

## 3. Results and Discussions

The quantity of active Mo species available at the substrate surface plays a pivotal role in the comprehensive growth process of MoS_2_, particularly in terms of nucleation and subsequent domain formation. Achieving a uniform distribution of gaseous-phase S is typically accomplished by maintaining a substantial distance between the S source and the growth substrate. In contrast, the Mo-containing source is typically positioned in close proximity to the growth substrate in order to ensure successful MoS_2_ growth. Consequently, this leads to the formation of a distinct gradient-like concentration distribution of the MoS_2_ nucleation sites on the substrate surface. This concentration gradient acts as a fundamental framework for dictating the final growth pattern, which typically presents as parabolic growth zones with varying morphologies, as illustrated in the schematic shown in Figure 1b. Figure 1c shows an SEM micrograph illustrating the transition from continuous films to individual domains in the outermost growth zones.

Numerical simulations, employing finite element modeling techniques conducted by R. A. Vila et al. [63], elucidated the behavior of the concentration gradient on the substrate surface as a function of the distance from the Mo source. The simulations revealed a gradual decrease in the concentration gradient, which is expected due to mixing and diffusion of the precursor in the ambient gas as it is pushed downstream by the argon carrier gas, corroborating the experimental observations of characteristic parabolic growth zones [25,30,37,46,50]. Moreover, the existence of such a concentration gradient instigates variations in the local Mo:S ratio along the length of the growth substrate, thereby augmenting the probability of diverse morphological evolutions [63], oxysulfide formations [34,37,49,60,61,62], and modifications in MoS_2_ domain shapes [25]. These findings underscore the significance of understanding and controlling the concentration gradient of active Mo species during MoS_2_ growth. The precise manipulation of this gradient can potentially be exploited to engineer and tailor the properties and characteristics of MoS_2_ nanostructures, thereby facilitating advancements in various technological applications.

### 3.1. Effect of Growth Temperature on MoS_2_ Growth

#### 3.1.1. Temperature Dependence

**Figure 2:**
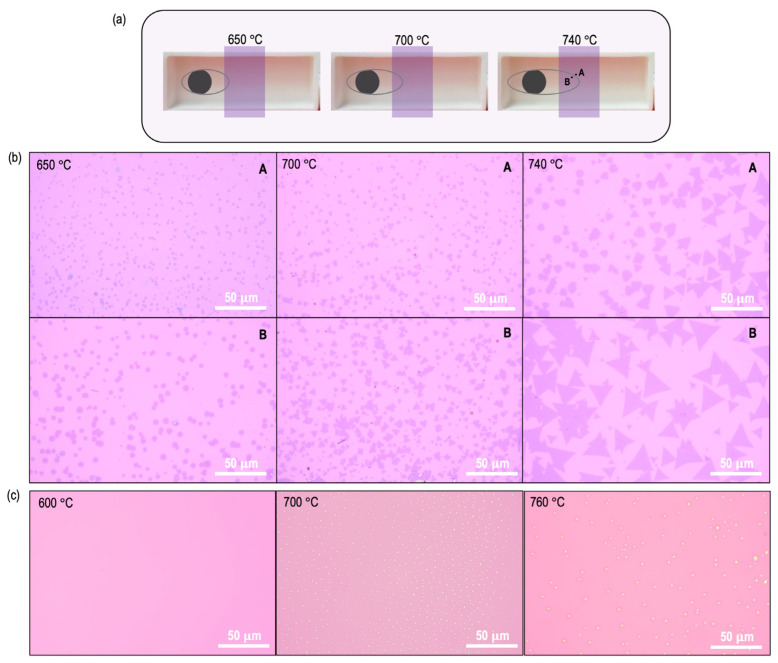
(**a**) Schematic depicting the increase in growth zones as a function of growth temperature. (**b**) Representative high-resolution optical images captured at indicated locations A and B in (**a**) for growth temperatures of 650 °C, 700 °C, and 740 °C. Locations A and B capture growth variations across the boundary of the outermost growth zone. (**c**) Optical images capturing nucleation density and seed size variation as the growth temperature is increased from 600 °C to 800 °C. Sulfur precursors were eliminated in this experiment. False color is used to make the individual seed size more visible.

**Figure 3:**
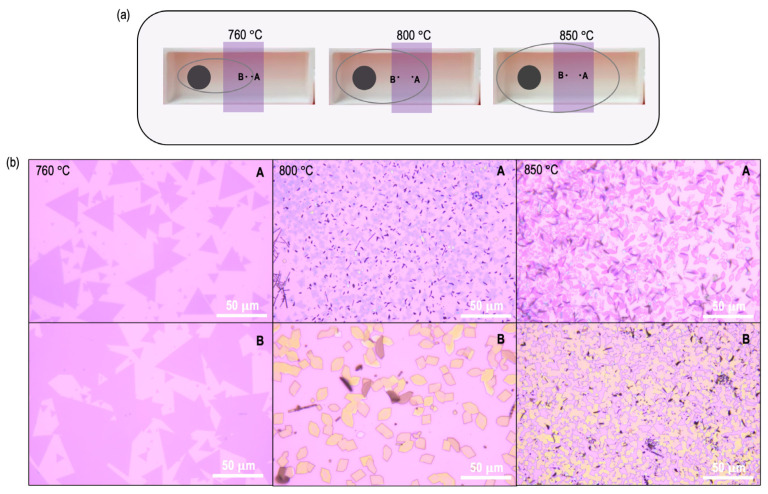
(**a**) Schematic depicting the dramatic increase in growth zones (grey oval) as the growth temperature is increased from 760 °C to 800 °C. (**b**) High-resolution optical images obtained at approximate locations around points A and B indicated in (**a**), for growth temperatures of 760 °C, 800 °C, and 850 °C, capturing the morphological evolution across these temperatures. Growth is typically absent below 650 °C. Optical images corresponding to growth results at 800 °C and 850 °C clearly indicate a shift to intermediate-state formation, while at 760 °C, ideal MoS_2_ domain size and density are obtained.

We began by first analyzing the MoS_2_ growth process at several different temperatures, ranging from 600 °C to 850 °C. The growth results are summarized in Figure 2 and Figure 3. In general, we consistently found no growth on the substrate when the growth temperature fell below 600 °C. This indicates that MoO_2_ exhibits a comparatively lower vaporization propensity than MoO_3_, which is well documented to vaporize even at temperatures as low as 350 °C. As the temperature is raised beyond 600 °C, MoS_2_ growth begins. Importantly, a systematic increase in growth zones, as shown in the schematics of Figure 2a and Figure 3a, was observed in the growth results.

Optical images captured for two closely spaced points (indicated in Figure 2a) that lie inside and outside the outermost growth zone are shown in Figure 2b for the temperatures of 650 °C, 700 °C, and 740 °C. It is clear from these images that at the lower temperatures of 650 °C and 700 °C, the grown MoS_2_ domain sizes are small, while the density is large. As the temperature increases to 740 °C, a clear deviation from this trend is observed, with an increase in domain size and a reduction in nucleation density. Optical images for the 600 °C condition, where no MoS_2_ growth occurs, were omitted from these results.

To delve deeper into the findings shown in Figure 2b, we investigated the impact of growth temperature on nucleation density and size, while deliberately omitting sulfur during the synthesis process. Figure 2c summarizes the results obtained at temperatures of 600 °C, 700 °C, and 760 °C. At 600 °C, it is clear that no nucleation sites exist. At 700 °C, the nucleation density appears to be large, while the individual seed sizes are very small. As the growth temperature is increased to 760 °C, two effects can be seen: a decrease in nucleation density, and a visible increase in seed size. These findings are consistent with the MoS_2_ growth results obtained at 760 °C (see Figure 3b), where we obtained large domain sizes, and the low nucleation density allows for the formation of continuous films with large grain-boundary separations (see Figure S2).

For the interested reader, a detailed investigation of grain-boundary separations at this temperature can be found in our previous study [21]. Shifting our attention now to the growth results obtained at 800 °C and 850 °C (Figure 3b), a stark deviation in the growth results is observed. At both of these temperatures, the central regions of the growth substrates are predominantly covered with intermediate states, and little-to-no MoS_2_ is grown here. Notably, this effect is much stronger at the highest temperature investigated here (850 °C). 

**Figure 4:**
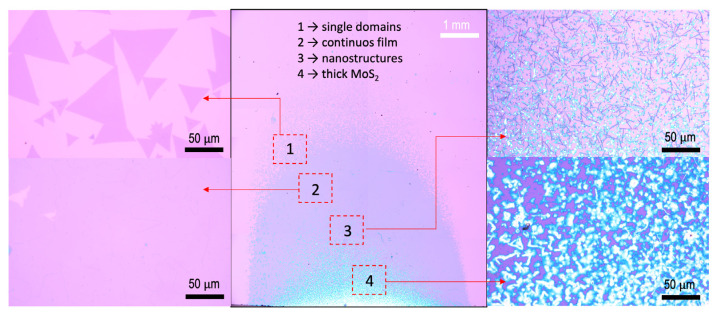
Detailed analysis of the growth results obtained at 760 °C. The center image corresponds to the high-resolution optically stitched image displaying the material grown on the entire substrate. Typical growth zones were obtained, similar to the schematic shown in Figure 1. The regions closest to the substrate edge are dominated by thick but pure MoS_2_ (bulk and nanostructures), while the morphology evolves to continuous MoS_2_ and individual triangular domains in the downstream regions, as indicated by high-magnification optical images displaying the morphology of materials grown in the regions marked 1–4. Detailed Raman line mapping of similar growth conditions may be obtained from our prior study [21].

To understand the correlation between the formation of intermediate states at temperatures above 800 °C and the absence of them at lower temperatures, we turned to a detailed analysis of the growth results obtained at 760 °C and 800 °C. To facilitate comprehensive visualization, we employed a high-resolution optical stitching approach, enabling the mapping of a centimeter-scale image encompassing the entire growth substrate. It is evident from the growth results obtained at 760 °C (see Figure 4) that the typical parabolic growth zones can be clearly observed.

The zones closest to the substrate edge are dominated by thick MoS_2_ and nanostructures. This is followed by a zone of continuous mm-scale film and, finally, the outermost zone consisting of individual MoS_2_ domains. Representative high-magnification optical images corresponding to regions 1–4 marked in the growth zones can be seen in Figure 4. The morphological variation between the various zones stems from the gradual change in the local S:Mo ratio along the gas flow direction [63]. 

**Figure 5:**
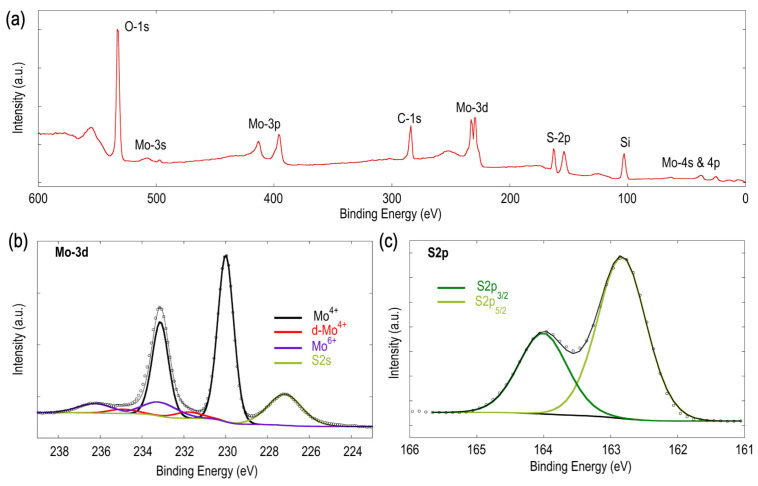
XPS spectra obtained for continuous MoS_2_ (region 2 of Figure 4): (**a**) Wide-spectrum scans revealing the presence of Mo and S signals. (**b**) Mo-3d and S2s core-level scans. (**c**) S-2p core-level scans.

X-ray photoelectron spectroscopy (XPS) was employed to analyze the chemical state of monolayer MoS_2_ films. The wide-spectrum scan shown in Figure 5a confirms the presence of Mo and S signals.

Additional peaks corresponding to oxygen (-O) and silicon (Si) were observed, likely originating from the SiO_2_/Si substrate. The C-1s peak stems from surface contamination. Figure 5b,c display high-resolution scans focusing on the Mo-3d and S-2p core-level spectra, respectively. The Mo-3d spectrum shows both the Mo-3d and S-2s core-level signals, which can be fitted with three Mo-3d doublets and one singlet peak (S-2s), respectively. The most intense Mo^4+^ doublet state (black trace) consists of two distinct peaks identified at 230.0 (3d_5/2_) and 233.12 eV (3d_3/2_), with a spin–orbit splitting (Δ) of approximately 3.12 eV. These peak positions closely match those reported for the Mo^4+^ oxidation state [64]. The absorption shoulder seen at 227.17 eV corresponds to the binding energy of the S-2s electron. The doublet peak located at 231.77 eV (red trace) stems from defect Mo^4+^ (d-Mo^4+^), corresponding to Mo atoms close to sulfur vacancies [65,66,67]. Finally, the doublet peak at the binding energy of 233.12 eV is due to the Mo^6+^ of Mo-O bonds [68,69,70] and can seemingly arise from various sources. It has been attributed to the presence of unreacted precursors [61,71], which are known to be a common source of contamination in CVD-grown MoS_2_. It has also been suggested that the Mo^6+^ states could be associated with surface oxidation from air exposure and cycling [72,73], and that these peaks are largely suppressed upon etching of samples for 10 s [73]. A further possibility is that the Mo^6+^ states originate from the formation of interfacial Mo-O bonds at the MoS_2_–SiO_2_ interface [74]. It is also important to consider the possibility of oxidation of MoO_2_ to MoO_3_ in the context of this study.

Examination of the sulfur chemical environment (Figure 5c) reveals a single doublet that can be resolved into the S 2p_3/2_ and 2p_1/2_ levels at 162.8 and 164.0 eV (Δ = 1.2 eV), respectively, indicating the presence of S_2_ states in MoS_2_ [65,66,75,76]. The absence of a higher binding energy doublet in the S-2p spectrum, the presence of a single S2s peak in the Mo-3d spectrum, and the very low d-Mo^4+^ contribution together indicate that effective sulfurization of the MoS_2_ has occurred [77].

The stoichiometry of the formed MoS_2_ material can be estimated by taking the ratios of the calculated atomic concentrations of the S-2p and Mo-3d components (see Table S1). This yields S:Mo values of ~1.7 and ~1.82, with and without including the d-Mo^4+^ contribution, respectively. These values are close to the value of ~1.86 reported for mechanically exfoliated MoS_2_ [58] and the statistically obtained average value of ~1.89 for MoS_2_ thin films reported in the literature [78]. Alternately, as outlined in the systematic study by Baker et al. [79], the MoS_2_ stoichiometry can also be determined to an accuracy of ±0.1 by measuring the distance between the S 2p_3/2_ and Mo 3d_5/2_ peaks. As shown in Table S1, we obtained a distance of 67.16 eV, which indicates a stoichiometric value of ~1.98 [67,79] for our sample. The contrasting methods make it challenging to use XPS to estimate the actual material’s stoichiometry, despite it being the most widely used composition analysis technique. Additionally, EDS mapping (Figure S3) was also performed for the continuous MoS_2_ and the bulk products. While the elemental maps of S and Mo confirm the presence of MoS_2_, the calculation of stoichiometry leads to erroneous results owing to the weak signal detection and the overlapping S and Mo energies [80,81], which is particularly problematic for the case of MoS_2_. Hence, this technique is not recommended for the reliable estimation of stoichiometry in monolayer and very-thin films of this material.

**Figure 6:**
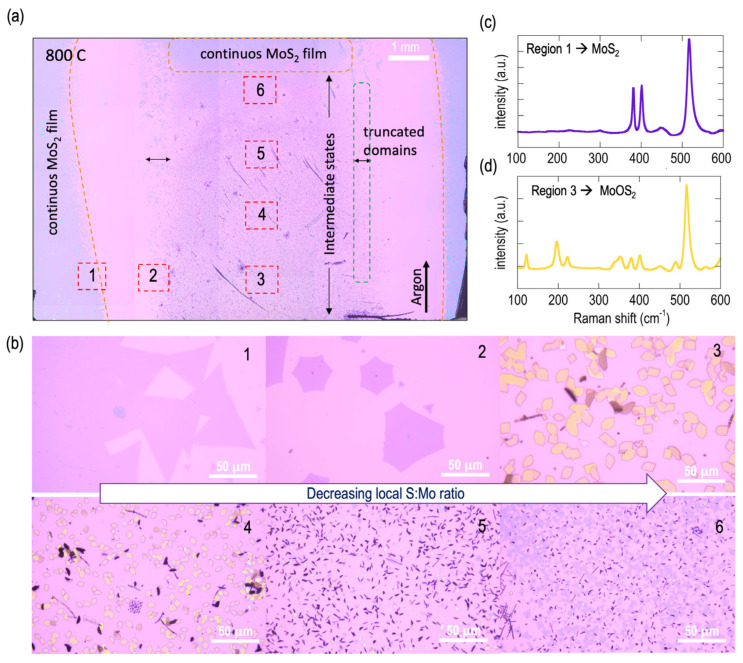
Detailed analysis of the growth results obtained at 800 °C: (**a**) High-resolution optically stitched image displaying the material grown on the entire substrate. The central regions of the substrate are dominated by intermediate states, while clean and continuous MoS_2_ grows towards the substrate edges and the downstream-most location of the substrate. The empty no-growth regions correspond to the area of the substrate overlapping the rim of the alumina crucible. (**b**) High-magnification optical images displaying the morphology of material grown in the regions labeled 1–6 in (**a**). (**c**,**d**) Raman traces obtained over a wide wavenumber range to highlight the difference in morphology at the central region of the wafer (predominantly MoOS_2_) vs. the edges (solely MoS_2_).

XRD analysis was conducted to gain further insights into the composition and phase of the materials grown on the substrate (refer to Figure S4 for details). The analysis confirmed the predominance of a pure 2H-MoS_2_ phase, aligning with findings from thin-film MoS_2_ grown using CVD [82]. Furthermore, the 2H MoS_2_ phase purity can be independently confirmed from the results of XRD, XPS (Figure 5), and Raman spectroscopy (see Figure 6), as they exhibit no related signals corresponding to the 1T-MoS_2_ phase [72,83,84]. XRD additionally revealed the presence of trace amounts of the precursor MoO_2_. We believe these to have arisen from unreacted precursors left behind upon cessation of CVD growth, as materials freeze at this stage and cannot be driven to complete reaction [85]. Overall, these observations are in reasonable agreement with the XPS findings.

Shifting our focus to the growth results achieved at 800 °C, it is evident from the optically mapped image in Figure 6a that a significant departure from the conventional parabolic growth zone pattern (as shown in Figure 4) has occurred.

The results reveal an interesting picture. Here, the majority of the middle region contains little-to-no visible growth of MoS_2_ and is instead largely dominated by intermediate states. Interestingly, continuous films of MoS_2_ now tend to grow on the left and right sides of the wafer. It must be noted that these growth regions lie exactly outside the rim of the alumina boat, representing the overhanging part of the substrate. Additionally, as indicated in Figure 6a, a region of continuous MoS_2_ is also found in the downstream-most location of the substrate. In the lateral direction and towards the end of the zone containing intermediate states, we can observe the formation of truncated MoS_2_ domains. Representative optical images capturing the various morphologies corresponding to the locations marked in Figure 6a are shown in Figure 6b. It can be seen from these images that the varying morphologies are closely related to their growth location on the substrate.

The results of wide-wavenumber Raman point analysis conducted in regions 1 and 3 are shown in Figure 6c,d, respectively. In Figure 6c, the two dominant Raman vibration modes that are characteristic of MoS_2_ can be seen. The E^1^_2g_ mode, corresponding to the in-plane vibration of S and Mo atoms, can be seen at a wavenumber of approximately 381.5 cm^−1^, while the A_1g_ mode, corresponding to the out-of-plane vibrations of S atoms, can be seen at about 400 cm^−1^. The resulting frequency difference (∆ω) between the two modes is therefore ~18.5 cm^−1^, confirming the monolayer nature of the grown MoS_2_ [86,87]. In contrast, as shown in Figure 6d, the Raman trace obtained from region 3 revealed the grown material to be intermediate MoOS_2_. While we have not pursued extensive line or area Raman mapping, simple optical inspection is usually sufficient to identify intermediate states that are known to have very distinct morphologies (rectangular domains) when compared to MoS_2_ and can therefore be easily identified on a substrate. 

It must be highlighted that, in contrast to our previous study [21], where we found that intermediate-state formation was largely quenched as long as the global S:Mo loading ratio was kept below the stoichiometrically required value of 3:1 required by the single-step VPS of MoO_2_, the appearance of intermediate states at higher temperatures in the present study, in spite of the global S:Mo loading ratio (20:1) being maintained well above the required 3:1 value, suggests a dramatic shift in the locally available S:Mo molar loading ratio at the higher growth temperatures. In order to explain this, let us consider the overhanging regions of the substrate where continuous films of MoS_2_ are formed. Here, the local S:Mo ratio should be large enough, and this may be possible due to the low availability of the Mo precursor at these locations. This is logical, as these locations are situated at the greatest lateral distance from the MoO_2_ source. On the other hand, driven by the high CVD temperature, the local Mo precursor available in the middle region of the substrate is substantially large in comparison, and this inevitably drives the local S:Mo ratio to very low values, thereby facilitating the formation of predominantly intermediate states. The formation of truncated domains in the regions indicated in Figure 6a suggests that the local S:Mo available here is just slightly below the stoichiometric requirement to fully form MoS_2_. This therefore indicates a decrease in the Mo concentration along the lateral direction of the growth zones. Along the direction of the gas flow, however, the decrease in the Mo concentration is more gradual, thereby leading to the slow morphological evolution seen in the captured images at locations 3–6 in Figure 6a. Here, one can see how the morphology evolves from predominantly intermediate MoOS_2_ growth at location 3 to a reduction in their sizes and the emergence of nanostructures at location 4. Continuing on to location 5, nanostructures dominate the growth, and further investigation of SEM images (Figure S5) reveals the formation of vertically-oriented structures in the mix. Finally, at location 6, small domains of MoS_2_ are predominant. As noted already, as we proceed further to the downstream-most location of the substrate, the morphology transitions to continuous films of MoS_2_, which simply means that the local S:Mo has increased enough to exit the regime of intermediate-state formation and enter the regime where the stoichiometric value is closer to or above 3:1. 

**Figure 7:**
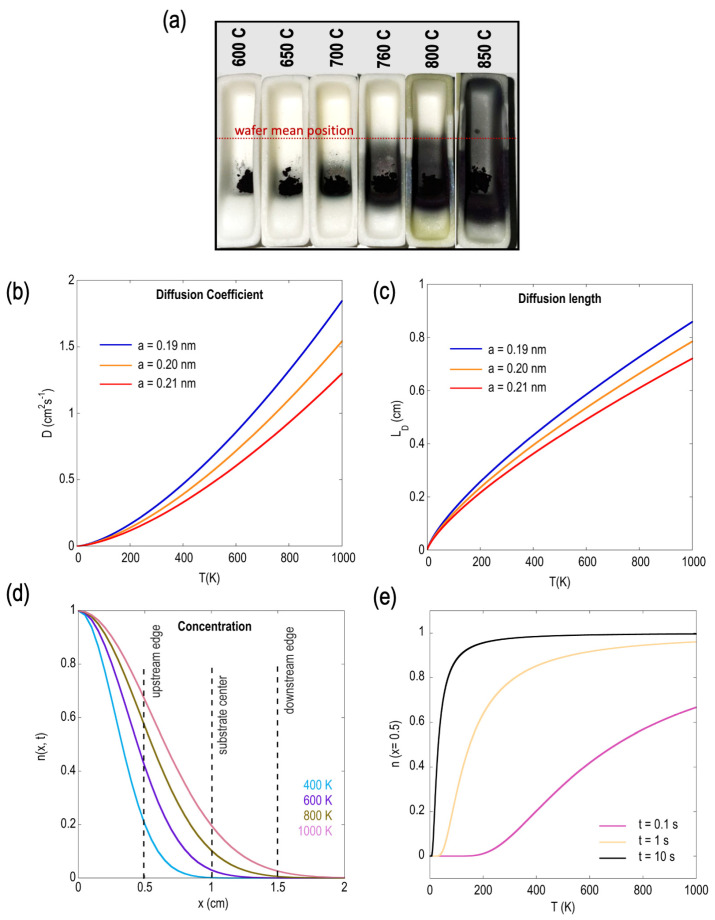
(**a**) Photograph of the alumina boat taken after growth was completed at the indicated growth temperatures. A dramatic change in precursor diffusion was noted at temperatures at and above 800 °C. Qualitative plots of the (**b**) diffusion coefficient and (**c**) diffusion length as a function of temperature at molecular sizes (a values) close to the expected value of MoO_2_ molecules. (**d**) Concentration profile vs. position from the precursor source, plotted using Equation (1), at a few indicated temperatures. (**e**) Concentration as a function of temperature at a few arbitrarily chosen residence time values.

The drastic differences in the growth outcomes at 760 °C and 800 °C may be further explained as a result of a dramatic increase in the diffusion behavior of the MoO_2_ precursor as the growth temperature is raised to and beyond 800 °C. Experimentally, this can be seen in the photograph shown in Figure 7a.

Here, we captured snapshots of the boat’s condition after the completion of the CVD growth process at each of the indicated temperatures. Clearly, the expansion of the black-shaded regions (which may be visualized as the region of precursor diffusion or the region where a concentration gradient exists) varies as the temperature is increased, with the effect seen to be pronounced at the highest temperatures.

In general, the diffusion of MoO_2_ vapor from the source to the substrate can be quantified by using a common solution to the diffusion equation [88]:

(1) $$n\left(x,t\right)=n\left(0,t\right)\;exp\left[-\frac{x^{2}}{4Dt}\right]\;$$

where *t* is the residence time of gases, *D* is the diffusion constant, and $n\left(0,t\right)$ and $n\left(x,t\right)$ are the concentrations of the gaseous species at the source and at a distance x away from the source, respectively. The unit of the diffusion constant is length^2^/second and is a function of temperature (*T*), pressure (*P*), and the radius of the precursor molecule (*a*). A rough estimate of *D* for a gas composed of hard spheres of equal size and mass is given as follows [88]:

(2) $$D\approx \sqrt{\frac{k^{3}}{\pi^{3}m}}\;\;\frac{T^{\frac{3}{2}}}{Pa^{2}}\;$$

where *k* is the Boltzmann constant. From Equation (2), if we assume a constant pressure of 1 atm (APCVD), it is evident that the diffusion constant increases as *T* increases. Another important parameter that relates to the diffusion constant and time is the diffusion length, *L_d_* = 2(*Dt*)^1/2^, and is understood as the characteristic length scale that obeys the diffusion equation. From this relation, one expects the diffusion length to also increase with temperature. These expected variations are evident in the plots shown in Figure 7b,c, where both *D* and *L_d_* values are plotted against temperature, respectively, within the range of values corresponding to the expected molecule size, *a*, of MoO_2_. The mass of the particles is calculated using knowledge of the molar mass (127.9 g/mol) of MoO_2_.

Using Equation (1), one can plot the variation in the concentration of available precursors at a distance *x* from the source. Assuming a short residence time (*t*) of 0.1 s and *n* (*x* = 0) = 1 gives us the concentration variation shown in Figure 7d, for several different temperatures, ranging from 400 to 1000 K. Two main conclusions can be drawn from the trends observed here: First, it is clear that the concentration of reacting MoO_2_ species at any given distance from the substrate increases with temperature. From the photograph shown in Figure 7a, we can note that the substrate position was fixed in all of our experiments at ~1 cm from the MoO_2_ source. The results of Figure 7d therefore suggest a significant reduction in the local S:Mo ratio as the growth temperature is raised, which explains the experimental observations of the strong formation of intermediate states at the highest temperatures of 800 °C and 850 °C. Second, it can also be seen in Figure 7d that, at higher temperatures, the concentration gradients can be strong and can extend along longer portions of the substrate. For example, from the 1000 K trace (pink), the concentration drops by nearly 70% across the length of the substrate. This explains the observation of larger growth zones and the morphologies transitioning from largely intermediate states in the bulk of the substrate to continuous films at the downstream-most edge of the substrate (see Figure 6a). These trends are further substantiated by the fact that at the highest growth temperature of 850 °C we found most of the growth substrate to be filled with nanostructures and intermediate states, while the overhanging portions of the wafer showed growth of truncated domains (Figure S6). While we did not perform detailed optical mapping of this case, the dramatic increase in the MoO_2_ precursor concentration and, hence, the low local S:Mo ratio, can be seen from the differences already noted from Figure 3b, as well as from the photograph shown in Figure 7a.

A note of caution is necessary regarding the variations presented in Figure 7b,c, as they are qualitative in nature and were plotted using a relatively short residence time to approximately mimic the experimental observations. A more robust quantitative analysis is not possible using Equation (1), since the powder vaporization of MoO_2_ occurs at much higher temperatures. We expect that more refined numerical simulations employing finite element analysis may be required for an accurate description of the actual diffusion dynamics. Having said that, it can be noted in Figure 7d, where we plotted concentration (at *x* = 1.5 cm) vs. temperature at a few different residence time values, that as the temperature increases the concentration increases rapidly at first, before saturating at higher values of *T*. This behavior is consistent with our experimental results, where we can see rapid changes in morphology within a short growth-temperature window.

A further point can be made as relates to the precursor type. For instance, considering the case of MoO_2_ vs. MoO_3_, while the diffusion equation would yield qualitative curves similar to those shown in Figure 7 for MoO_3_, the actual diffusion behavior could be vastly different for the two precursors when considering that the powder vaporization of these precursors occurs at very different temperatures. Consequently, this will result in completely different concentration profiles across the substrate and, hence, different S:Mo precursor ratios available for growth. The difference in reaction pathways between the two precursors can also be viewed as important, because the VPS of MoO_3_ to MoS_2_ is a multistep process, as opposed to the single-step VPS of MoO_2_. Experimental validation of these assumptions can be found in Figure S7 of the Supplementary Materials, where we show how, under the exact same experimental conditions, the morphology of the material grown on the substrate when using MoO_3_ is in striking contrast to that obtained when using MoO_2_ precursors. These findings therefore suggest that the experimental parameters necessary for obtaining ideal MoS_2_ growth could vary vastly depending on the choice of Mo precursor source. 

#### 3.1.2. Effect of Varying MoO_2_ Precursor Amount/Formation of Multilayers

**Figure 8:**
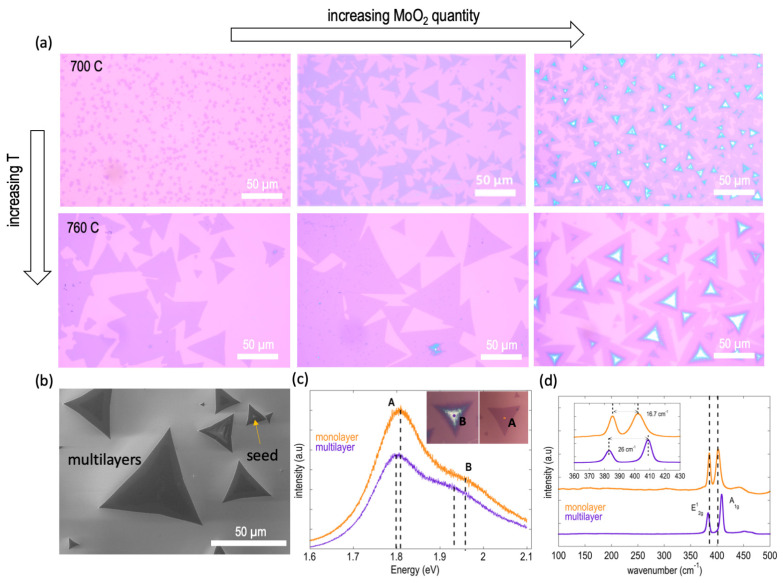
Effect of varying the MoO_2_ precursor quantity: (**a**) Optical images capturing the formation of multilayer MoS_2_ as the MoO_2_ precursor quantity is increased from 2 mg to 5 mg to 10 mg. The upper and lower panels correspond to the growth results obtained at 700 °C and 760 °C, respectively. (**b**) SEM image showing multilayer formation and seeds. (**c**) Photoluminescence (PL) trace showing the characteristic A and B excitonic peaks for single-layer and multilayer/bulk MoS_2_. (**d**) Wide-wavenumber Raman spectra showing the characteristic E^1^_2g_ and A_1g_ peaks of MoS_2_ and the absence of low-wavenumber peaks corresponding to intermediate states. Inset shows the calculated peak difference values.

In this section, we focus on the lower temperatures, where intermediate states are absent, and we explore the effect of changing the MoO_2_ precursor quantity. The results obtained at growth temperatures of 700 °C and 760 °C are shown in Figure 8a, from which it is evident that while the increase in the MoO_2_ quantity results in larger MoS_2_ domain sizes, it also initiates the formation of multilayers.

The effect is pronounced at the highest MoO_2_ quantity of ~10 mg, as evident from the multilayer formation even at the lower temperature of 700 °C, where the domain sizes are small. These findings are consistent with Equation (1), where it can be seen that the concentration increases proportionally to the precursor concentration set at *x* = 0. Upon further inspection of the optical images shown in Figure 8a, it can be seen that the additional layers grow from the center of the MoS_2_ flake, which results from the increased MoO_2_ concentration. The presence of seeds and the grown multilayers can be seen more clearly in the SEM micrographs shown in Figure 8b.

In Figure 8c, we show an example photoluminescence (PL) spectrum of the monolayer and multilayer domains. The presence of the characteristic A and B direct excitonic transitions at approximately 1.81 and 1.95 eV, respectively, in the monolayer MoS_2_ domain, along with the notable intensity of the A peak, serves to indicate both the monolayer nature and the excellent crystalline quality of the grown MoS_2_ [2,17]. Conversely, when examining spectra obtained from the multilayer domain, a decrease in the photoluminescence (PL) intensity is observed, accompanied by a slight redshift in the excitonic transitions. These results are consistent with the observed characteristics exhibited by both CVD-grown and exfoliated MoS_2_ layers, as reported in previous studies [89,90,91]. The main panel of Figure 8d shows Raman spectra corresponding to the monolayer and multilayer MoS_2_ domains, obtained over a wide wavenumber range. The two dominant Raman vibration modes that are characteristic of MoS_2_ can be seen here, and the absence of any intermediate-state formation is proven by the absence of additional peaks in the low-wavenumber region. The E^1^_2g_ mode, corresponding to the in-plane vibration of S and Mo atoms, is seen at a wavenumber of approximately 385.4 cm^−1^, while the A_1g_ mode, corresponding to the out-of-plane vibrations of S atoms, is seen at about 402 cm^−1^ (see the inset in Figure 8d). The resulting frequency difference (∆ω) between the two modes is therefore ~16.7 cm^−1^, confirming the monolayer nature of the MoS_2_ domain [86,87]. In agreement with the literature [86], the peak difference obtained for the multilayer/bulk MoS_2_ was found to be ~26 cm^−1^.

### 3.2. Role of Substrate Position 

**Figure 9:**
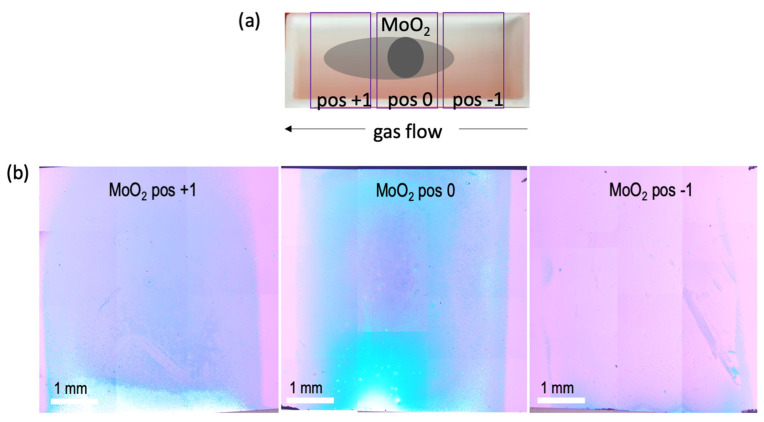
(**a**) Schematic setup used to study the effect of varying substrate position with respect to the Mo precursor used. (**b**) Optical stitched images capturing the formation of MoS_2_ growth zones for positions +1 cm, 0 cm, and −1 cm from the MoO_2_ source.

We can obtain further insights on the diffusion of precursors by studying the role of the substrate position. Due to the role of gas flow driving the reactants, it is expected that a larger diffusion zone will be formed downstream from the precursor location than in the upstream direction. The schematic shown in Figure 9a illustrates this effect.

As a result, the variation in the substrate position should result in growth zones following this pattern. This is evident in Figure 9b, where we show the growth results obtained through optical image stitching at a growth temperature of 760 °C and at three different substrate positions, labeled −1 cm, 0 cm, and +1 cm. The zero position corresponds to the situation where the substrate is placed exactly over the precursor location. At this position, as expected, the majority of the substrate is covered with thick MoS_2_ material, which corresponds to the white- and blue-colored regions on the substrate. At the position +1 cm, the classic parabolic zones emerge, with each zone exhibiting varying morphological characteristics. 

Based on the above observations, it is perceivable that increasing the sample height in the experiment should lead to growth behavior mimicking that obtained when changing the horizontal position of the substrate. By applying the diffusion equation in the vertical direction, one should expect a systematic reduction in the Mo precursor concentration with sample height, and this should, in principle, allow for the growth of more homogenous MoS_2_ thin films at an experimentally determined optimal height. While the specific experiments to reveal the expected growth–height dependence were not conducted in this study, the insights obtained may help improve our understanding of the growth process. We should note that under the experimental conditions used in the current study, diffusion-limited growth predominated, as evidenced by the distinct parabolic zone growth pattern. To obtain homogenous and continuous MoS_2_ films that are large-scale (or even wafer-scale), it would be necessary to modify the experimental parameters such that growth can happen outside the diffusion-limited regime. As indicated in the literature, several strategies may be employed, including the reduction in Mo precursor quantity [55], space confinement [56], increasing the substrate–source gap height [26], using barriers [49], or employing a multisource tube furnace that allows for more uniform Mo precursor concentration available at the growth substrate [48]. In a previously published work [21], we have also shown how changing the MoO_2_ precursor distribution on the boat can dramatically alter the zonal pattern and lead to better substrate coverage. 

Having said that, in order to gain a comprehensive understanding of the diffusion processes involved in the CVD growth of MoS_2_ using MoO_2_, further investigation is needed, particularly in the context of low-pressure chemical vapor deposition (LPCVD). Equation (2) clearly suggests that the diffusion coefficient varies much more strongly with pressure than with temperature. Reducing the pressure to 1 Torr, for instance, can dramatically increase the diffusion length and, hence, drive the reaction out of the diffusion-limited regime. Additionally, the influence of the gas flow rate and sulfur precursor quantity on diffusion and the formation of growth zones should be carefully considered. Our preliminary findings suggest a potential dependency, as evidenced by the varying extent of the observed parabolic growth zones in relation to these parameters. However, the present results are inconclusive, highlighting the necessity for more extensive experiments. These forthcoming investigations will be part of our future work, aiming to provide deeper insights into these aspects of diffusion phenomena.

## 4. Conclusions

We conducted a detailed study of the effect of growth temperature in the single-step VPS of MoS_2_ using direct MoO_2_ precursors. In general, our experiments revealed the diffusion of MoO_2_ to be a strong function of temperature, as evidenced by two key findings: the dramatic enlargement of growth zones, and the strong morphological changes observed across the growth substrate. By varying the temperature in a wide range of 600–850 °C, we were able to identify important growth criteria for the formation of large-scale MoS_2_. For the given set of experimental conditions investigated in this study, we found that at temperatures below 650 °C, no MoS_2_ growth occurred, which we attributed to the high vaporization temperature of MoO_2_ and, hence, the absence of nucleation. Between temperatures of 650 and 700 °C, growth commenced, but the yield of MoS_2_ was small due to the small growth zones. At 760 °C, expanded growth zones allowed us to see the morphological change from thick MoS_2_ to nanostructures at the upstream location of the substrate, and to mm-scale continuous monolayer MoS_2_ and individual domains in the downstream regions. At the downstream locations, the domain growth was characterized by lower density and large sizes, which coalesced to form continuous monolayer MoS_2_ with large grain-boundary separations. Increasing the MoO_2_ precursor quantity revealed the formation of multilayer MoS_2_ in the outermost growth zones. The observed morphological evolutions from thick MoS_2_ to nanostructures to MoS_2_ can be understood to be a result of the increasing local S:Mo ratio along this direction.

Raising the growth temperature to 800 °C revealed dramatic changes in the morphology, with intermediate states dominating the CVD growth, and the various morphologies obtained could be associated with the expected local S:Mo variations in those regions. By performing a qualitative analysis of the diffusion equation, we showed how the diffusion coefficient and the concentration gradient are strong functions of temperature, and how they can be utilized to interpret the experimental observations. Finally, we also studied the dependence of substrate-to-MoO_2_-precursor distance and found the growth zone formation to be consistent with expectations. When directly comparing the growth of MoS_2_ using MoO_2_ and MoO_3_ precursors, we observed significant differences in the morphology of the grown materials. We attributed these findings to the differences in diffusion behavior and vaporization temperatures, in addition to significant differences in the reaction pathways associated with these precursors.

In conclusion, although additional research is necessary to gain a complete understanding of the characteristics and applications of MoO_2_ precursors for producing pristine and large-scale MoS_2_, we firmly believe that the findings presented in this study will provide a foundation for the successful adoption of MoO_2_ as a precursor in the commercial growth of MoS_2_, ensuring both high quality and safety standards.

## Data Availability

The data presented in this study are available upon request from the corresponding author. The data are not publicly available due to legal/ethical reasons.

## Supplementary Materials

The following supporting information can be downloaded at: https://www.mdpi.com/article/10.3390/ma16134817/s1, Figure S1: Growth temperature profiles; Figure S2: MoS_2_ domain size and grain-boundary separation; Figure S3: Elemental Mapping results; Figure S4: XRD results; Figure S5: Morphology evolutions at 800 °C; Figure S6: Morphology evolutions at 850 °C; Figure S7: Precursor comparison; Table S1: XPS Quantification- Determination of Stoichiometry.
