# Supplementary material for: CVD Synthesis of MoS2 Using a Direct MoO2 Precursor: A Study on the Effects of Growth Temperature on Precursor Diffusion and Morphology Evolutions
Source: Materials (Basel). 2023 Jul 4;16(13):4817. doi: 10.3390/ma16134817 (PMC10343541; doi:10.3390/ma16134817)
Supplement: Supplementary file 1 [file materials-16-04817-s001.zip › materials-2452239-supplementary.pdf]

Supplementary Information

CVD Synthesis of MoS_2 Using Direct MoO_2 Precursor: A Study on the Effects of Growth Temperature on Precursor Diffusion and Morphology Evolutions

Ratchanok Somphonsane [1,2], Tinna Chiawchan [1], Waraporn Bootsa-ard [1] and Harihara Ramamoorthy [3,*]

1 Department of Physics, School of Science, King Mongkut's Institute of Technology Ladkrabang, Bangkok 10520, Thailand; ratchanok.so@kmitl.ac.th (R.S.); 62605032@kmitl.ac.th (T.C.); 62050723@kmitl.ac.th (W.B.-a.)
2 Thailand Center of Excellence in Physics, Commission on Higher Education, 328 Si Ayutthaya Road, Bangkok 10400, Thailand
3 Department of Electronics Engineering, School of Engineering, King Mongkut's Institute of Technology Ladkrabang, Bangkok 10520, Thailand
* Correspondence: harihara.ra@kmitl.ac.th

1. Growth temperature profiles

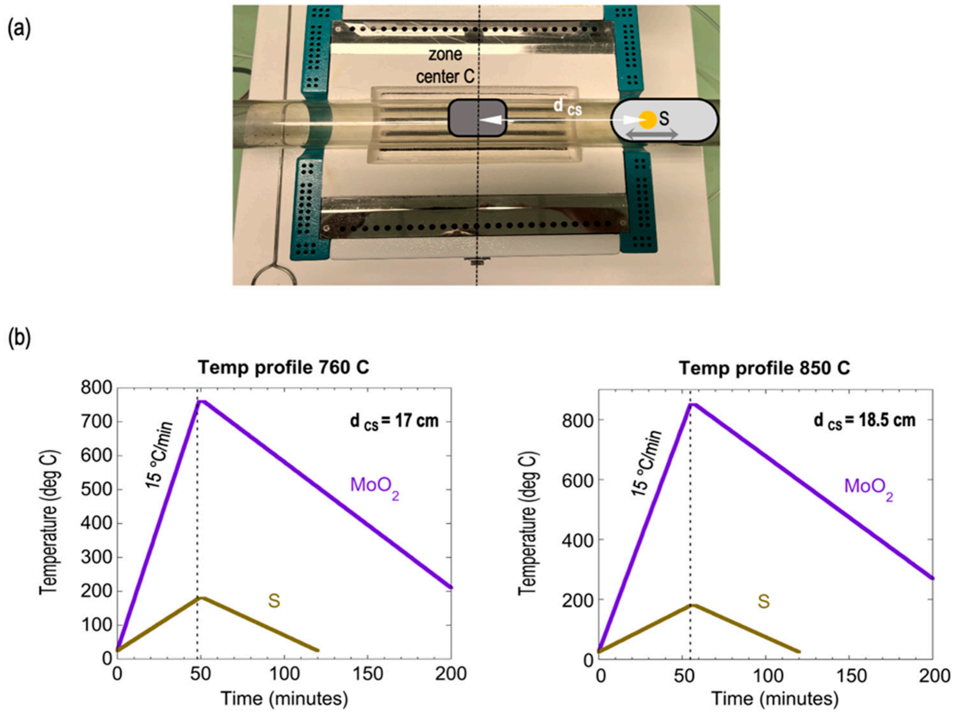

Figure S1. (a) Photograph of CVD setup. MoO_2 precursors and the growth substrate (separated by ~ 1 cm) are positioned exactly at the center of the heating zone (length 22 cm). The distance d_{CS} of the Sulphur from the zone center is 17 ± 1.5 cm depending on the set MoS_2 growth temperature, so as to achieve an approximately fixed S zone temperature of 180 °C in all growth experiments. For experiments conducted at 650 °C, 760 °C and 850 °C, d_{CS} ~ 15.5 cm, 17 cm and 18.5 cm, respectively. These values are calibrated prior to conducting the actual growth experiments by externally measuring the crucible temperature using an infrared temperature sensor while the furnace is held at the respective temperatures. (b) Temperature profile of the MoO_2 zone and the expected profiles for the S zone, shown for the cases of growth at 760 °C and 850 °C.

2. MoS_2 domain size and grain-boundary separation

(a)

(b)

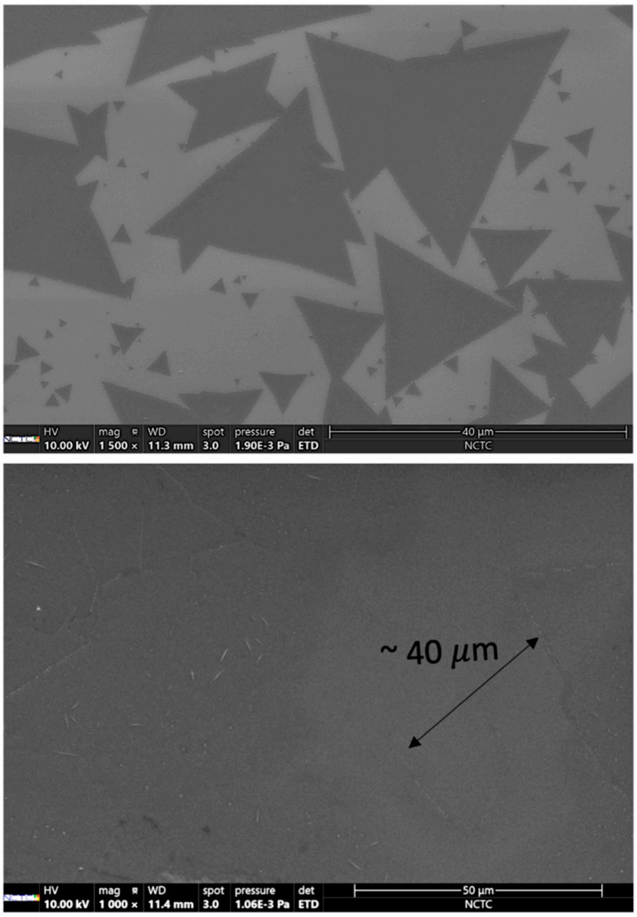

Figure S2. SEM images of (a) MoS_2 domains and (b) continuous MoS_2 found at the outermost growth zones for the experiment conducted at the ideal growth temperature of 760 °C. The large domain sizes (~30 – 40 μm) are achieved due to the low gas flow rate setting (10 sccm). In (b), grain boundaries are visible in the continuous films and their separations can be seen to be as large as 40 μm, which indicates good film quality.

Table S1. XPS Quantification- Determination of Stoichiometry: XPS quantification data containing peak binding energies and FWHMs of detected components as well as percentage atomic concentrations calculated from the relative peak areas, taking into consideration the system-defined Relative sensitivity factors (RSF). As per the general rule, stoichiometry is determined by using the S2p transition as the integrated peak area is larger in comparison to the S2s transition.

	**BE [eV]**	**FWHM [eV]**	**Integrated peak area [eV. counts/s]**	**RSF**	**Normalized peak area [eV. counts/s]**	**Atomic conc. [%]**
S 2s	227.17	1.92	6268.72	0.39		
S 2p 3/2	162.83	0.85	6083.92	0.67	9080.5	39.40
S 2p 1/2	164.01	0.85	3041.9	0.67	4540.1	19.70
Mo 3d (4+) 5/2	229.99	0.90	15672.34	3.32	4720.6	20.48
Mo 3d (4+) 3/2	233.12	0.93	9161.44	3.32	2759.5	11.97
Mo 3d (d-4+) 5/2	231.77	1.55	1006.9	3.32	303.28	1.31
Mo 3d (d-4+) 3/2	234.90	1.55	681.67	3.32	205.32	0.89
Mo 3d (6+) 5/2	233.12	1.95	2843.01	3.32	856.33	3.71
Mo 3d (6+) 3/2	236.25	1.95	1921.908	3.32	578.89	2.51
**Total**					**23045**	**100**
S2p: $Mo3d^{4+}$ = 1.82						
S2p: $Mo3d^{4+,d4+}$ = 1.7						

3. Elemental Mapping results

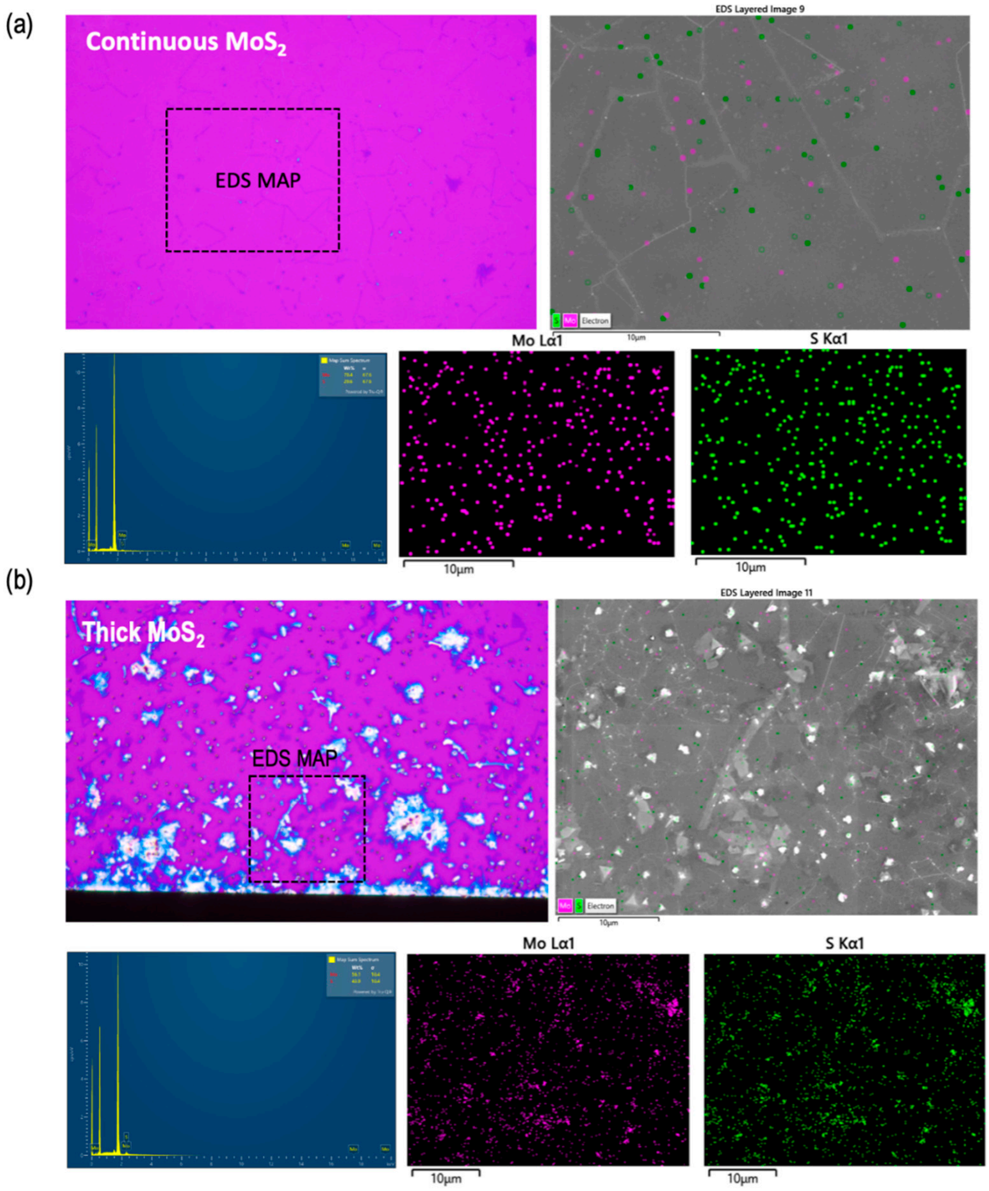

Figure S3. Elemental mapping (EDS) results obtained from (a) continuous MoS_2 formed in outer zones and (b) Thick MoS_2 product formed near the wafer edge. Elemental maps of Mo and S confirm the presence of MoS_2. Stoichiometric ratio from EDS have not been reported in this work as they are erroneous, owing to the very weak signal (in comparison to Si and O) as well as overlapping Mo and S energies, which is a known issue.

4. XRD results

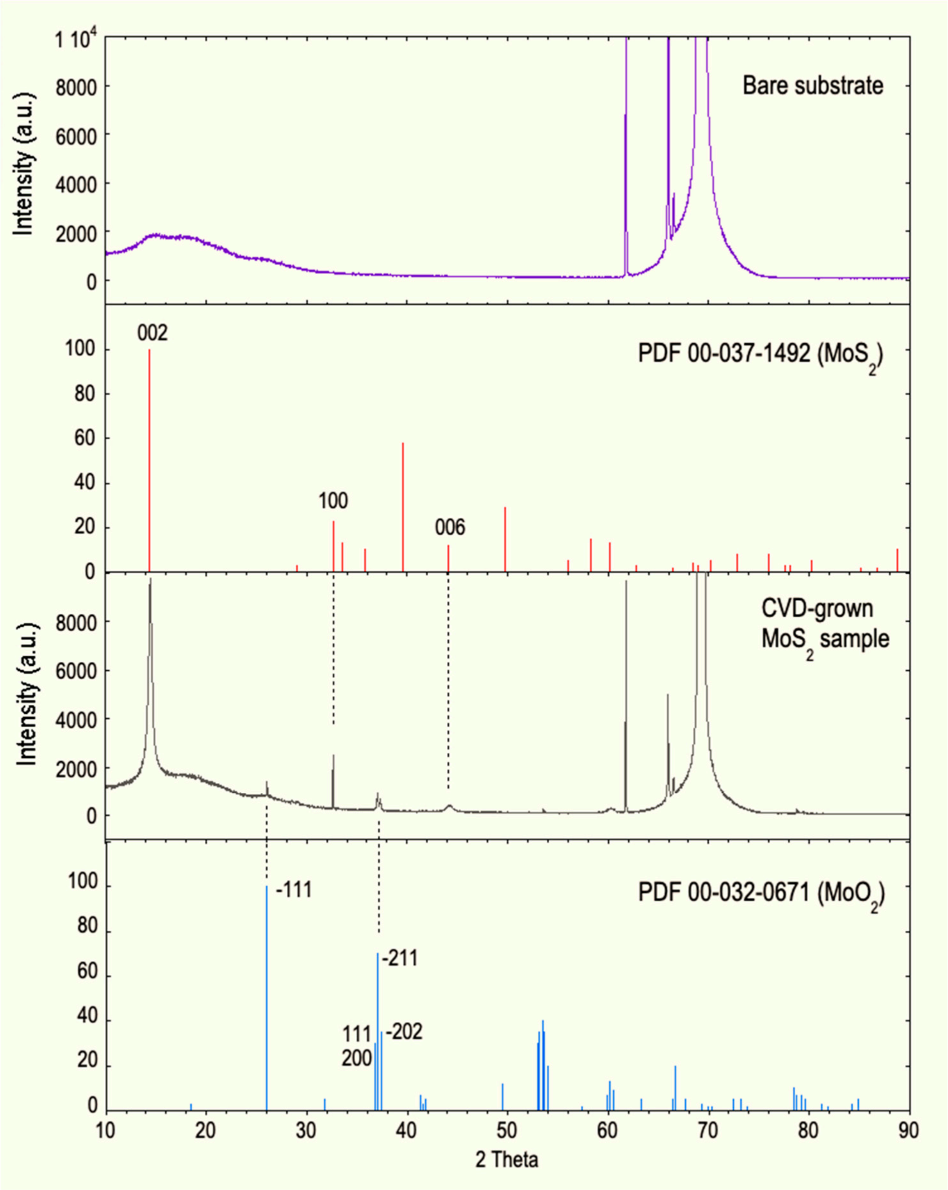

Figure S4. XRD spectra obtained from the CVD-grown MoS_2 sample grown at 760 °C. XRD spectrum of the bare substrate (Si/300 nm SiO_2) was also collected which allowed for a clearer analysis of the MoS_2 diffraction peaks. All observed peaks are compared against XRD database of MoS_2 (PDF # 00-037-1492) and MoO_2 (PDF # 00-032-0671), the precursor used for MoS_2 growth in this study. Two main diffraction peaks are detected at 2θ positions of 14.378° and 32.677°, and correspond respectively to the (002) and (100) lattice planes of 2H-MoS_2 phase. The 002 orientation is dominant as evidenced by the comparatively large intensity. In addition, weak second-order reflections of the (006) plane is also detected at 2θ position of 44.152°. Absence of other higher order peaks corresponding to the bulk MoS_2 reference are not detected, which may imply the overall thin film nature of the materials. In addition to the MoS_2 peaks, clear but relatively small intensity reflections that correspond to the reference peaks of MoO_2 are detected at the 2θ position of 18.45° (-111) and a collection of closely spaced reflections (200, 111, -211, -202) are also detected at a mean position ~37°. We believe these to arise from trace amounts of unreacted precursor left behind upon cessation of CVD growth, as materials freeze at this stage and cannot be driven to complete reaction. The lower value (20:1) of S: Mo loading ratio used in this study in comparison to our previous study (100:1), may also be a contributing factor. The parabolic growth zone pattern suggests that the majority of these products may be present at the wafer edge.

5. Morphology evolutions at 800 °C

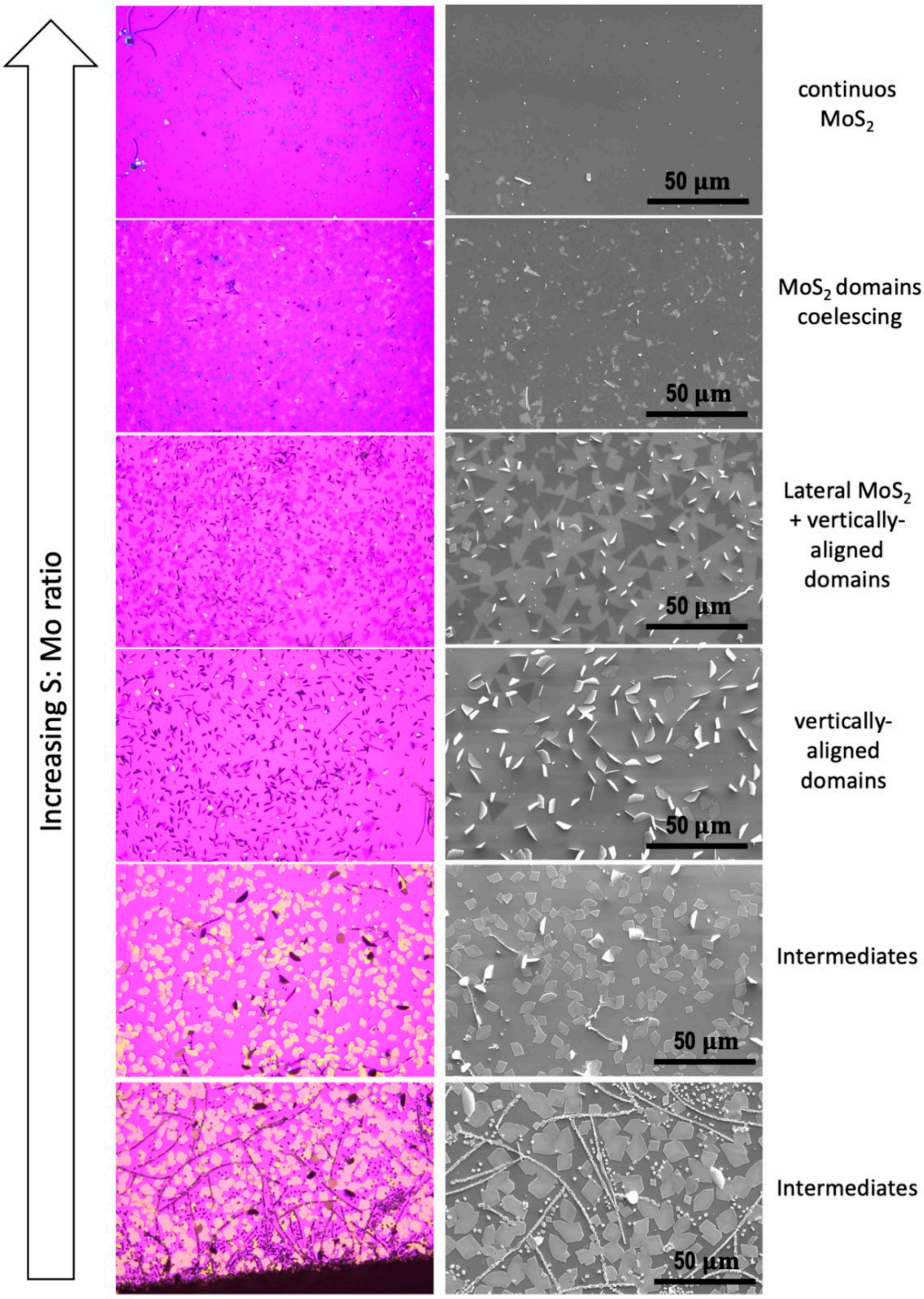

Figure S5. SEM images capturing varying growth morphology at 800 °C. The morphology is seen to evolve from intermediates to MoS_2 with transformations in between. Just prior to the formation of lateral MoS_2 domains, the dominance of vertically-oriented structures are also observed in the SEM micrographs.

6. Morphology evolutions at 850 °C

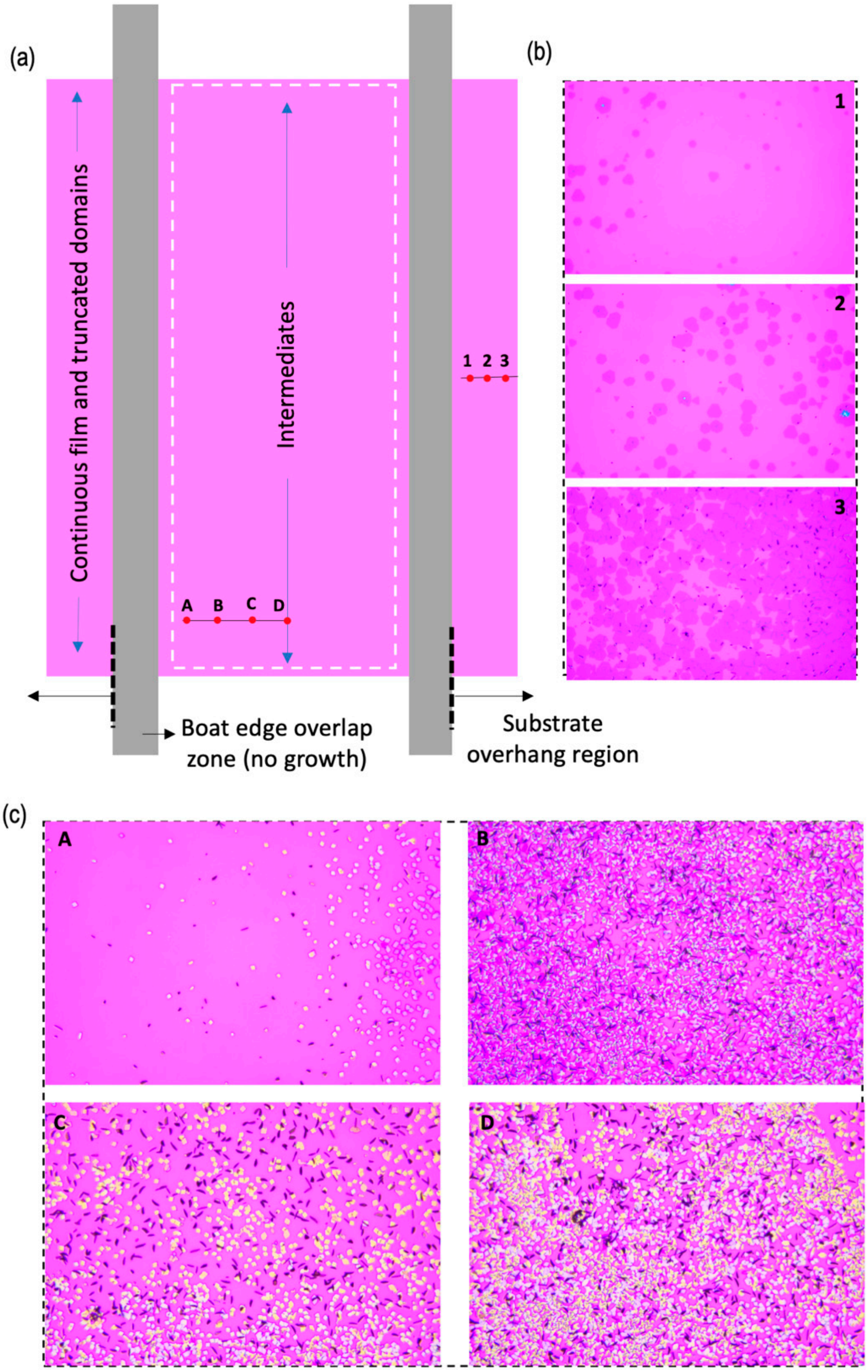

Figure S6. General results of growth morphologies at the highest investigated growth temperature of 850 °C. Substrate is largely comprised of intermediate states, while the overhanging regions of the substrate show the formation of truncated MoS_2 domains and continuous films forming from the coalescence of the individual domains.

7. Precursor comparison

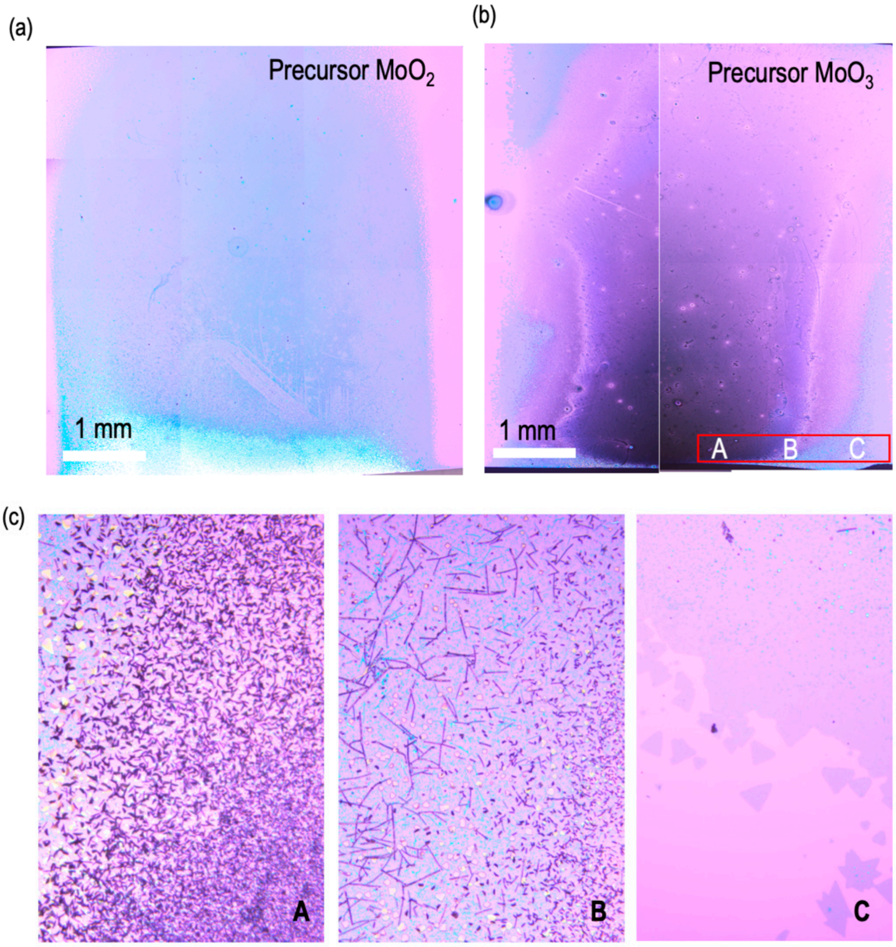

Figure S7. A direct comparison of MoS_2 growth obtained from using either (a) MoO_2 or (b) MoO_3 as the precursor. A gas flow rate of 30 sccm is used for this experiment. Clear differences in growth pattern and morphologies are noted, which can be explained as a result of dramatic difference in diffusion behaviour of these two precursor materials. These could arise from the significantly different vaporization temperatures as well as the different reaction pathways followed to form MoS_2. These differences could imply the reason behind the requirement of large S: Mo loading ratios used for achieving ideal MoS_2 growth from MoO_3, as is often reported in the literature.
